# Supplementary material for: Observation of Bloch oscillations dominated by effective anyonic particle statistics
Source: Nat Commun. 2022 May 2;13:2392. doi: 10.1038/s41467-022-29895-0 (PMC9061765; doi:10.1038/s41467-022-29895-0)
Supplement: Supplementary file 1 — Supplementary information [file 41467_2022_29895_MOESM1_ESM.pdf]

Supplementary Information: Observation of Bloch oscillations dominated by effective anyonic particle statistics

W. Zhang et al.

Supplementary Note 1. Details for the derivation of eigen-equation for the 2D circuit simulator with $\theta = \pi$.

Supplementary Note 2. The influence of the value of C_e on the correspondence between the eigen-spectra of 2D circuit simulators and 1D two-anyon models.

Supplementary Note 3. The influence of the finite size effect on impendence spectra of designed circuit simulators.

Supplementary Note 4. Numerical results of the Bloch oscillation based on the 1D lattice model of two anyons.

Supplementary Note 5. Simulating the anyonic Bloch oscillation with different excitation frequencies, external forces and grounding capacitors C_e.

Supplementary Note 6. The influence of disorder effects on the Bloch oscillations and required phase distributions in circuit simulators.

Supplementary Note 7. The Influence of loss effect on the simulation of anyonic Bloch oscillations by designed circuit simulators.

Supplementary Note 8. Numerical results of Bloch oscillations based on the 1D anyonic lattice model with dissipations.

Supplementary Note 9. The precise correspondence between the time-dependent Schrödinger equation of two bosons and two pseudofermions and the designed RC circuit simulators.

Supplementary Note 10. Numerical results of anyonic Bloch oscillations beyond bosons and pseudofermions.

Supplementary Note 1. Details for the derivation of eigen-equation for the 2D circuit simulator with $\boldsymbol{\theta = \pi}$. In this section, we give a detailed derivation of the circuit eigen-equation, which can be mapped to the 1D stationary Schrödinger equation of two pseudofermions. Here, each lattice site possesses two circuit nodes. In this case, the voltage and current at the site (m, n) should be written as $V_{(m,n)}$ =[$V_{(m,n),1}$, $V_{(m,n),2}$]^T and $I_{(m,n)}$=[$I_{(m,n),1}$, $I_{(m,n),2}$]^T. The voltage on the circuit node (m, n) is in the form of $V_{(m,n),j}e^{i\omega t}$ (j=1, 2).

First, we focus on the node pair located at the diagonal line (n, n) of the circuit. Carrying out Kirchhoff's law on the circuit node pair (n, n), we obtain the following equation as

$$\begin{vmatrix} I_{(n,n),1} \\ I_{(n,n),2} \end{vmatrix} = i\omega^{-1}\{-\frac{1}{L}\begin{vmatrix} 1 & -1 \\ -1 & 1 \end{vmatrix}\begin{vmatrix} V_{(n,n),1} \\ V_{(n,n),2} \end{vmatrix} + \omega^2 C \begin{vmatrix} V_{(n,n),1} - V_{(n-1,n),1} \\ V_{(n,n),2} - V_{(n-1,n),2} \end{vmatrix} + \omega^2 C \begin{vmatrix} V_{(n,n),1} - V_{(n+1,n),1} \\ V_{(n,n),2} - V_{(n+1,n),2} \end{vmatrix}$$

$$+\omega^2 C \begin{vmatrix} V_{(n,n),1} - V_{(n,n-1),2} \\ V_{(n,n),2} - V_{(n,n-1),1} \end{vmatrix} + \omega^2 C \begin{vmatrix} V_{(n,n),1} - V_{(n,n+1),2} \\ V_{(n,n),2} - V_{(n,n+1),1} \end{vmatrix} + \omega^2 (n+n) C_F \begin{vmatrix} V_{(n,n),1} \\ V_{(n,n),2} \end{vmatrix} + \omega^2 C_e \begin{vmatrix} V_{(n,n),1} \\ V_{(n,n),2} \end{vmatrix}\},$$

(1)

where C_e and $(n+n)C_F$ are grounding capacitances at node (n, n). C is the capacitance used for connecting circuit nodes belonging to adjacent lattice sites. L is the inductor used for linking a pair of circuit nodes belonging to the same lattice site. We assume that there is no external source, so that the current flowing out of the node is zero. In this case, Supplementary Eq. (1) becomes:

$$\frac{1}{\omega^2 L}\begin{vmatrix} 1 & -1 \\ -1 & 1 \end{vmatrix}\begin{vmatrix} V_{(n,n),1} \\ V_{(n,n),2} \end{vmatrix} = [4C + C_e + 2nC_F]\begin{vmatrix} V_{(n,n),1} \\ V_{(n,n),2} \end{vmatrix} - C\begin{bmatrix} 0 & 1 \\ 1 & 0 \end{bmatrix}\left(\begin{vmatrix} V_{(n,n-1),2} \\ V_{(n,n-1),1} \end{vmatrix} + \begin{vmatrix} V_{(n,n+1),2} \\ V_{(n,n+1),1} \end{vmatrix}\right) -$$

$$C\begin{vmatrix} V_{(n-1,n),1} \\ V_{(n-1,n),2} \end{vmatrix} - C\begin{vmatrix} V_{(n+1,n),1} \\ V_{(n+1,n),2} \end{vmatrix}. \quad (2)$$

Performing the diagonalization of Supplementary Eq. (2) with a unitary transformation

$$F = \frac{1}{\sqrt{2}}\begin{bmatrix} 1 & e^{i\pi} \\ 1 & -e^{i\pi} \end{bmatrix}, \quad (3)$$

Supplementary Eq. (2) becomes

$$\frac{1}{\omega^2 L}\begin{vmatrix} 0 & 0 \\ 0 & 2 \end{vmatrix}\begin{vmatrix} V_{\uparrow,(n,n)} \\ V_{\downarrow,(n,n)} \end{vmatrix} = [4C + C_e + 2nC_F]\begin{vmatrix} V_{\uparrow,(n,n)} \\ V_{\downarrow,(n,n)} \end{vmatrix} - C\begin{bmatrix} 1 & 0 \\ 0 & e^{-i\pi} \end{bmatrix}\begin{vmatrix} V_{\uparrow,(n,n-1)} \\ V_{\downarrow,(n,n-1)} \end{vmatrix}$$

$$-C\begin{bmatrix} 1 & 0 \\ 0 & e^{i\pi} \end{bmatrix}\begin{vmatrix} V_{\uparrow,(n,n+1)} \\ V_{\downarrow,(n,n+1)} \end{vmatrix} - C\begin{vmatrix} V_{\uparrow,(n-1,n)} \\ V_{\downarrow,(n-1,n)} \end{vmatrix} - C\begin{vmatrix} V_{\uparrow,(n,n+1)} \\ V_{\downarrow,(n,n+1)} \end{vmatrix} \quad (4)$$

New bases are written as $V_{\uparrow(\downarrow),(m,n)}$=$F[V_{(m,n),1}, V_{(m,n),2}]^{\mathrm{T}}$, which are two decoupled terms acting as a pair of pseudospins $V_{\uparrow,(m,n)} = (V_{(m,n),1} + V_{(m,n),2})/\sqrt{2}$ and $V_{\downarrow,(m,n)} = (V_{(m,n),1} - V_{(m,n),2})/\sqrt{2}$. Thus, Supplementary Eq. (4) can be divided into two independent equations as:

$$0 = [4C + C_e + (n+n)C_F]V_{\uparrow,(n,n)} - C(V_{\uparrow,(n,n-1)} + V_{\uparrow,(n,n+1)} + V_{\uparrow,(n-1,n)} + V_{\uparrow,(n,n+1)}), \quad (5)$$

$$\frac{1}{\omega^2 L/2}V_{\downarrow,(n,n)} = [4C + C_e + 2nC_F]V_{\downarrow,(n,n)} - C(e^{-i\pi}V_{\downarrow,(n,n-1)} + e^{i\pi}V_{\downarrow,(n,n+1)} + V_{\downarrow,(n-1,n)} + V_{\downarrow,(n,n+1)}).$$

(6)

Following the same derivation discussed above, we can write the eigen-equation at every circuit node (m, n). In particular, as for circuit nodes satisfying n=m-1, the corresponding eigen-equation is given by

$$\frac{1}{\omega^2 L/2}V_{\downarrow,(n,n-1)} = [4C + C_e + 2nC_F]V_{\downarrow,(n,n-1)} - C(V_{\downarrow,(n,n-2)} + e^{i\pi}V_{\downarrow,(n,n)} + V_{\downarrow,(n-1,n-1)} + V_{\downarrow,(n+1,n-1)}). \quad (7)$$

Moreover, as for the case of n=m+1, we have,

$$\frac{1}{\omega^2 L/2}V_{\downarrow,(n,n+1)} = [4C + C_e + 2nC_F]V_{\downarrow,(n,n+1)} - C(e^{-i\pi}V_{\downarrow,(n,n)} + V_{\downarrow,(n,n+2)} + V_{\downarrow,(n-1,n+1)} + V_{\downarrow,(n+1,n+1)}). \quad (8)$$

The remained circuit nodes satisfy the relationship of $|n - m| > 1$, and we have,

$$\frac{1}{\omega^2 L/2}V_{\downarrow,(m,n)} = [4C + C_e + 2nC_F]V_{\downarrow,(m,n)} - C(V_{\downarrow,(m,n-1)} + V_{\downarrow,(m,n+1)} + V_{\downarrow,(m+1,n)} + V_{\downarrow,(m-1,n)}). \quad (9)$$

Combing Supplementary Eqs. (6)-(9), the eigen-equation of the designed circuit simulator is described by:

$$(f_0{}^2/f^2 - 4 - C_e/C)V_{\downarrow,mn} = -e^{-i\pi(\delta_{m,n}+\delta_{m,n+1})}V_{\downarrow,m(n+1)} - e^{i\pi(\delta_{m,n}+\delta_{m+1,n})}V_{\downarrow,m(n-1)}$$

$$-V_{\downarrow,(m+1)n} - V_{\downarrow,(m-1)n} + (m+n)(C_F/C)V_{\downarrow,mn}. \quad (10)$$

We provide the following identification of tight-binding parameters in terms of circuit elements as:

$$J = 1,\ F = \frac{C_F}{C},\quad \varepsilon = \frac{f_0{}^2}{f^2} - 4 - \frac{C_e}{C},\quad f_0 = \frac{1}{2\pi\sqrt{CL/2}}, \quad (11)$$

where J, F and ε correspond to the strength of particle hopping, the external forcing and the eigen-energy of two anyons. In this case, Supplementary Eq. (10) becomes

$$\varepsilon c_{mn} = -J[e^{i\theta(\delta_{m,n}+\delta_{m+1,n})}c_{m(n-1)} + e^{-i\theta(\delta_{m,n}+\delta_{m-1,n})}c_{m(n+1)} + c_{(m-1)n} + c_{(m+1)n}] + F(m+n)c_{mn} \quad (12)$$

with c_{mn} corresponding to the voltage pseudospin $V_{\downarrow,(m,n)}$. It is noted that Supplementary Eq. (12) is consistent with the eigen-equation for the 1D two-pseudofermion system (Eq. (4) in the main text).

Supplementary Note 2. The influence of the value of C_e on the correspondence between the eigen-spectra of 2D circuit simulators and 1D two-anyon models. It is known that the appearance of Bloch oscillations depends on the equally spaced eigen-spectrum of two bosons and two pseudofermions, and the BO periods are determined by the associated energy-level spacings. However, due to the nonlinear relationship between the eigen-frequency of the circuit simulator and the eigen-energy of two anyons $f = f_0/(\varepsilon + 4 + C_e/C)^{1/2}$, the distribution of the frequency-spectrum for the circuit simulator should

not be equally spaced as the two-anyon counterpart.

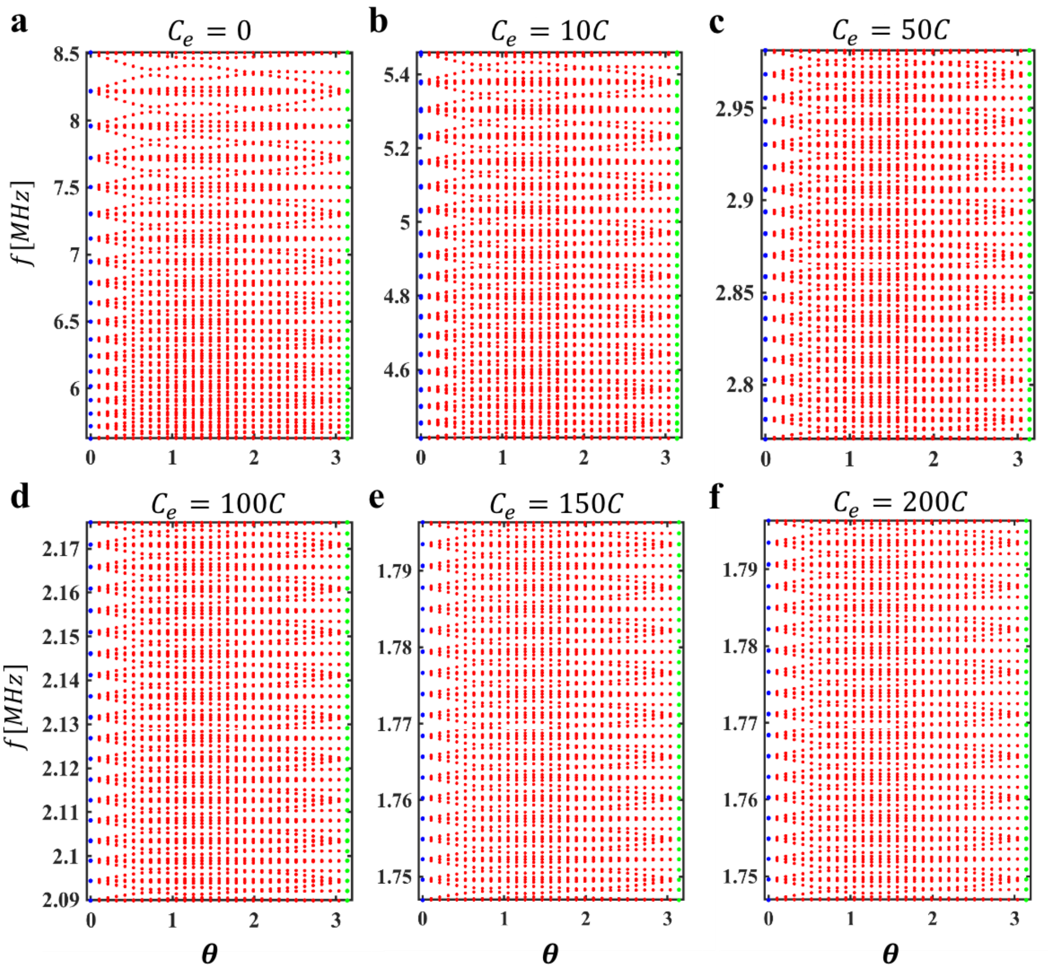

Supplementary Figure 1. The influence of the value of C_e on the eigen-spectra of 2D circuit simulators. (a)-(f) The calculated eigen-frequencies of designed circuit simulators as a function of the statistical angle θ with $C_e = 0$, $C_e = 10C$, $C_e = 50C$, $C_e = 100C$, $C_e = 150C$, and $C_e = 200$C, respectively.

In this part, we show that such a deviation could become negligible by setting a uniform grounding with a large value of C_e in the designed circuit simulator. As shown in Supplementary Figures 1(a)-1(f), we plot the eigen-frequency of the designed circuit simulator as a function of the statistical angle θ with $C_e = 0$, $C_e = 10C$, $C_e = 50C$, $C_e = 100C$, $C_e = 150C$, and $C_e = 200$C, respectively. It is clearly shown that the eigen-spectrum with $C_e = 0$ is not equally spaced for the circuit simulator at $\theta = 0$ and $\theta = \pi$, where the frequency gap gets increased as the corresponding eigen-frequency. By increasing the value of C_e, the frequency gaps in the higher- and lower-frequency ranges trend to a constant. In our designed circuit simulator, we set $C_e = 200C$. In this case, we can see that the nearly equal-spaced

frequency-spectra appear in the circuit simulators with $\theta = 0$ ($\Delta f_B \approx 1862.57Hz$) and $\theta = \pi$ ($\Delta f_f \approx 931.28Hz$), respectively. With such a good correspondence between the frequency-spectrum of the designed 2D circuit and the energy-spectrum of the 1D two-anyon model, the behavior of quantum statistics-dominated BO can be effectively implemented by the designed circuit simulator.

Supplementary Note 3. The influence of the finite size effect on impendence spectra of designed circuit simulators. In this section, we will show that the equal-space impedance peaks could exist in a much wider frequency range when the size of the circuit simulator is increased. As shown in Supplementary Figure 2a (and Supplementary Figure 2b), we calculate the summed impedance, where the summed circuit nodes are selected on diagonals of two-boson (and two-pseudofermion) circuit simulator from (14,14) to (28, 28). Here, the size of designed circuits is set as N=41, that is larger than that used in Fig. 2a of the main text. In addition, it is noted that a uniform grounding capacitor ($18C_F$) should be deleted from each node in the larger circuit to ensure the same onsite potential at the central node of two circuits with different sizes. We can see that the at low- and high-frequency ranges, the larger circuit simulator could still exhibit equally spaced impedance peaks. This is due to the fact the boundary states have negligible responses under the excitation of circuit nodes at (14,14) and (28, 28), making only the equally spaced bulk state be excited.

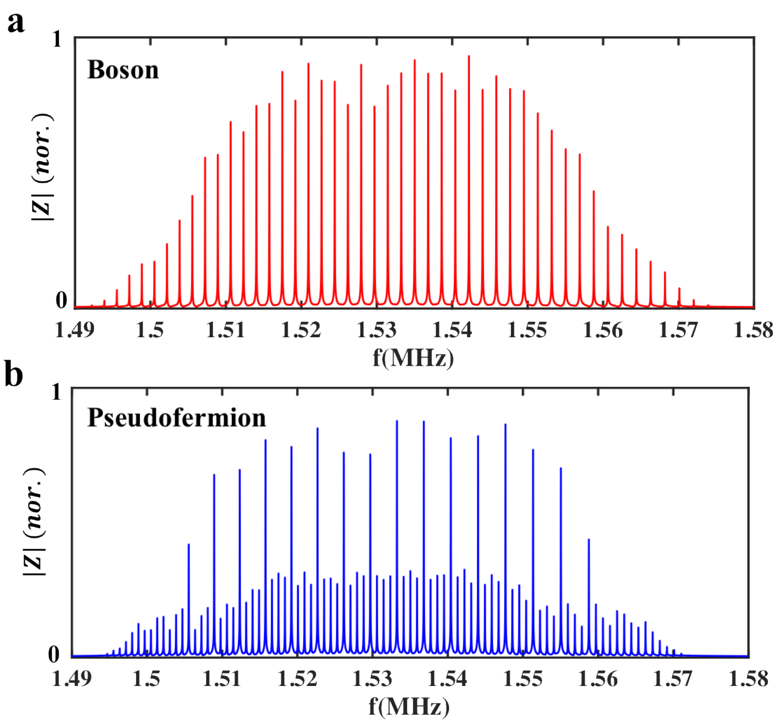

Supplementary Figure 2. The simulated impendence spectra of circuit simulators with N=41. (a)

and (b) The summed impedance of two-boson and two-pseudofermion circuit simulators.

Supplementary Note 4. Numerical results of the Bloch oscillation based on the 1D lattice model of two anyons. In this part, we give numerical results of BOs described by the 1D lattice model of two anyons with N=23. The evolution equations for the probability amplitude c_{mn} with the input two-anyon excitation $\psi_{\mathrm{in}}(\mathrm{t})$ is expressed as:

$$i\partial_t c_{mn} = -J[e^{i\theta(\delta_{m,n}+\delta_{m+1,n})}c_{m(n-1)} + e^{-i\theta(\delta_{m,n}+\delta_{m-1,n})}c_{m(n+1)} + c_{(m-1)n} + c_{(m+1)n}] + F(m+n)c_{mn} + \psi_{\mathrm{in}}(\mathrm{t})c_{mn}. \tag{13}$$

To observe the BO of two bosons and pseudofermions, the two-anyon excitation is set as:

$$\psi_{\mathrm{in}}(\mathrm{t}) = \delta_{m,12}\delta_{n,12}e^{i\varepsilon t} \tag{14}$$

with other parameters being $\varepsilon = 20$, J=1 and F=0.5, respectively.

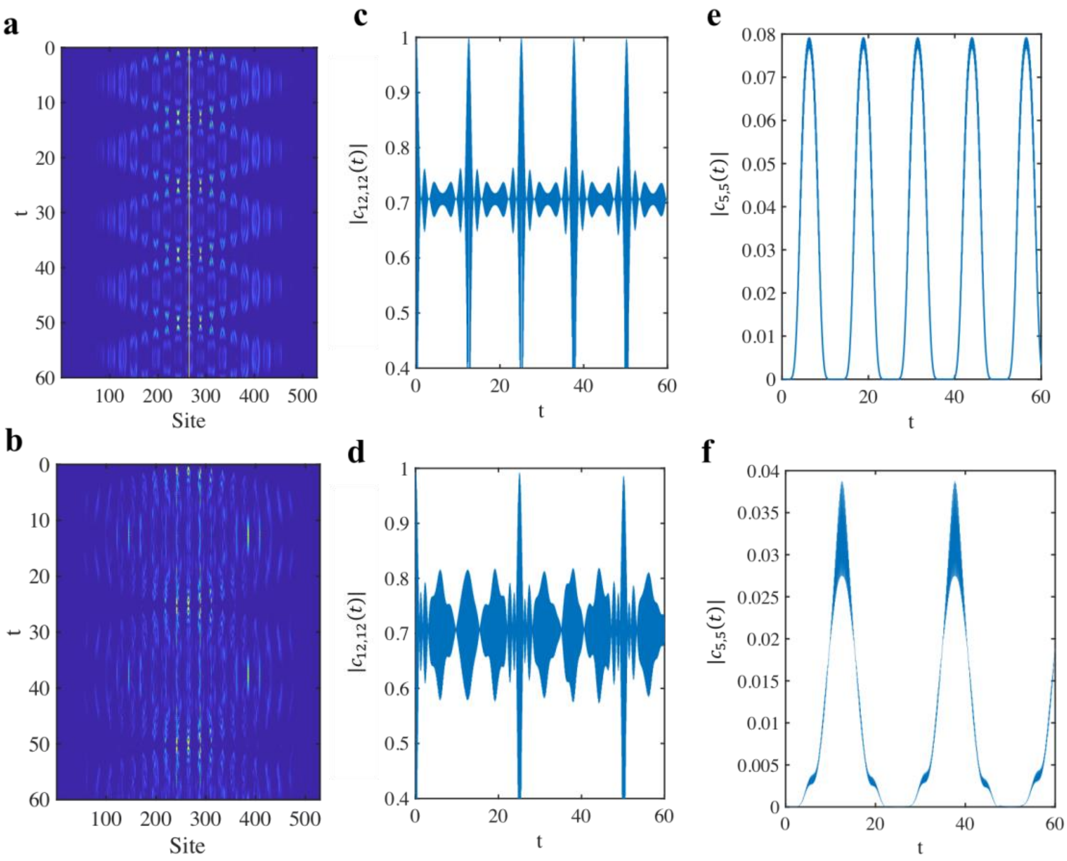

Supplementary Figure 3. Numerical results of the Bloch oscillation for two anyons with F=0.5. (a) and (b) The evolution of $|c_{mn}(t)|^2$ with $\theta = 0$ and $\theta = \pi$, respectively. (c) and (d) display

the evolution of $|c_{12,12}(t)|$ with $\theta = 0$ and $\theta = \pi$. Here, the external force is set as *F*=0.5.

As shown in Supplementary Figures 3a and 3b, we calculate the evolution of $|c_{mn}(t)|^2$ with $\theta = 0$ and $\theta = \pi$. Supplementary Figures 3c and 3e display the evolution of fixed states $|c_{12,12}(t)|$ and $|c_{5,5}(t)|$with $\theta = 0$, respectively. And, Supplementary Figures. 3d and 3f display the evolution of fixed states $|c_{12,12}(t)|$ and $|c_{5,5}(t)|$with $\theta = \pi$, respectively. It is clearly shown that periodic breathing dynamics of both two bosons and two pseudofermions appear, and the oscillation period of the two bosons is almost twice of that for two pseudofermions, being consistent with the calculated Wannier-Stark spectra in Fig. 1d.

Then, we focus on the anyonic BOs with a smaller external force *F*=0.3. Supplementary Figures 4a and 4b present the calculated evolutions of $|c_{mn}(t)|^2$ with $\theta = 0$ and $\theta = \pi$. Moreover, in Supplementary Figures 4c and 4d, the numerical results for the evolution of $|c_{12,12}(t)|$ with $\theta = 0$ and $\theta = \pi$ are calculated. We can see that the BO frequency related to a pair of pseudofermions ($\theta = \pi$) is still half of that for two bosons ($\theta = 0$). Additionally, the BO period and amplitude are all increased when the external force is decreased.

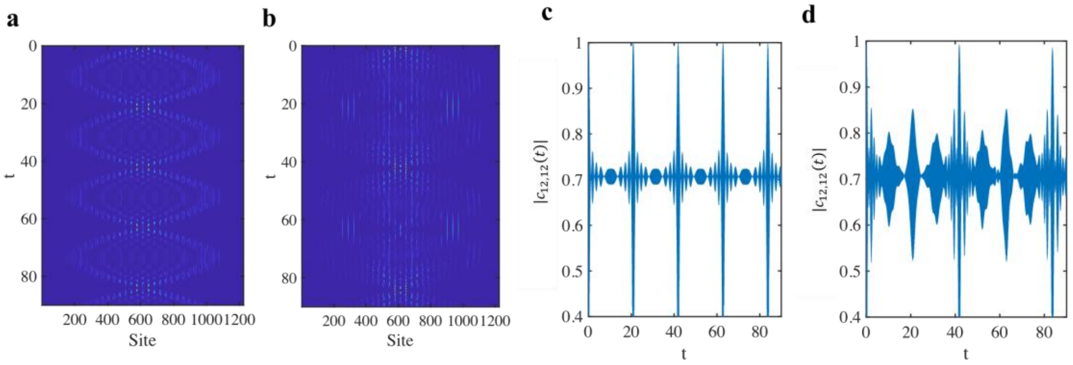

Supplementary Figure 4. Numerical results of the Bloch oscillation for two anyons with F=0.3. (a) and (b) The evolution of $|c_{mn}(t)|^2$ with $\theta = 0$ and $\theta = \pi$ in the absence of particle interactions, respectively. (c) and (d) display the evolution of the particle density function of $|c_{12,12}(\mathrm{t})|$ with $\theta = 0$ and $\theta = \pi$. Here, the external force is set as *F*=0.3.

Supplementary Note 5. Simulating the anyonic Bloch oscillation with different excitation frequencies, external forces and grounding capacitors C_e. First, we perform circuit simulations of BOs with different values of C_e. As shown in Supplementary Figures 5a-5d, we calculate the time-dependent evolution of pseudospin $|V_{\downarrow,[m,n]}(t)|^2$ at each node in the 2D circuit simulator (the left chart corresponds to $\theta = 0$ and the right chart corresponds to $\theta = \pi$) with $C_e = 0$, $C_e = 0.5nF$, $C_e = 2nF$

and $C_e = 4nF$, respectively. Here, the excitation frequency is set as 1.56MHz and other parameters are the same as those used in Fig. 2. We can see that the larger the value of C_e is, the more ideal periodic BOs appear. This is due to the fact that the nearly perfect frequency-spectrum with equally spaced frequency gaps could only be realized by setting an extremely large value of C_e, as demonstrated in Supplementary Figure 1.

Then, we simulate anyonic Bloch oscillations with a different external force by our designed electric circuits, that is, $C_F = 3pF$. Before circuit simulations, we calculate the evolution of two anyon eigen-energies as a function of θ with *J*=1 and *F*=0.3, as shown in Supplementary Figure 6a. The eigen-frequencies of the designed circuit simulators with $C_e = 0$, $C_e = 50C$, and $C_e = 200\mathrm{C}$ are shown in Supplementary Figures. 6(b)-6(d). It is shown that the frequency-spectrum of the circuit simulator is consistent with that of two anyons with a large value of C_e. In particular, we have $\Delta f_B \approx 1130Hz$ and $\Delta f_f = 565Hz$.

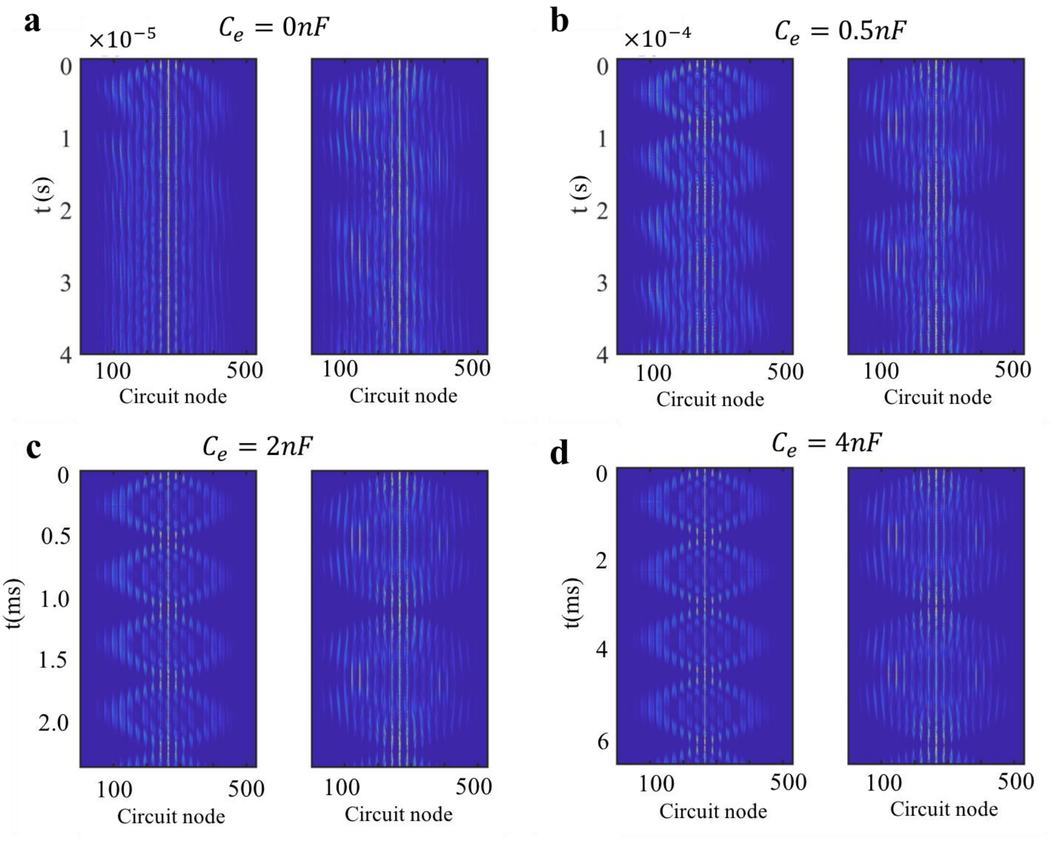

Supplementary Figure 5. Simulated results of Bloch oscillation with different grounding capacitors C_e of the circuit. (a)-(d) The time-dependent evolution of pseudospin $|V_{\downarrow,[m,n]}(t)|^2$ at each node in the

circuit simulator with $C_e = 0$, $C_e = 0.5nF$, $C_e = 2nF$ and $C_e = 4nF$.

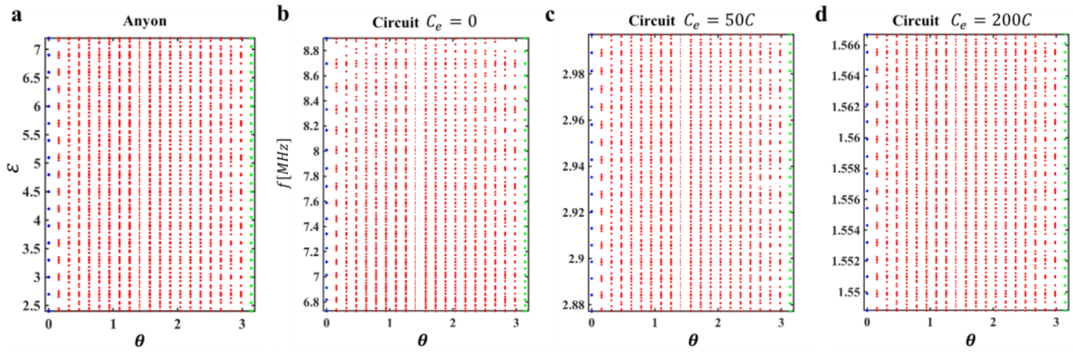

Supplementary Figure 6. The eigen-spectra of circuit simulators with different C_e. (a). The evolution of two-anyon eigen-energies as a function of θ with J=1 and F=0.3. (b)-(d) The eigen-frequencies of circuit simulators ($C_F = 0.3C$) with $C_e = 0$, $C_e = 50C$, and $C_e = 200$C.

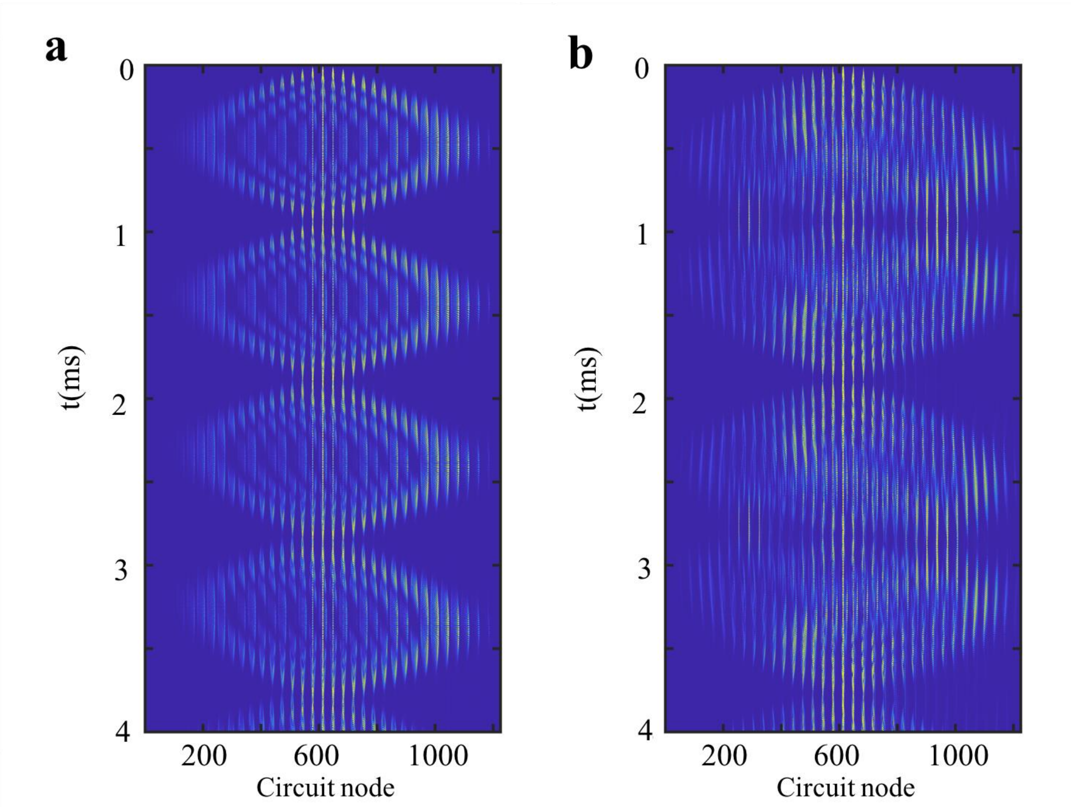

Supplementary Figure 7. Simulated results of Bloch oscillation with $C_e = 200$C and $C_F = 0.3C$. The time-dependent evolution of pseudospin $|V_{\downarrow,[m,n]}(t)|^2$ at each node in the circuit simulator ($C_e =$

200C and $C_F = 0.3C$) with $\theta = 0$ for (a) and $\theta = \pi$ for (b).

Next, we calculate the time-dependent evolution of pseudospin $|V_{\downarrow,[m,n]}(t)|^2$ at each node in the 2D circuit simulator ($C_e = 200$C and $C_F = 0.3C$) with $\theta = 0$ and $\theta = \pi$, as shown in Supplementary Figures 7a and 7b. Here, the excitation frequency is set as *f*=1.511 MHz, and the voltage pseudospin is excited by setting the input signal as $[V_{(12,12),1} = V_0 e^{i2\pi ft}, V_{(12,12),2} = -V_0 e^{i2\pi ft}]$ with $V_0 = 1V$. It is shown that the absolute value of voltage pseudospin displays the periodic breathing dynamics for both conditions. Moreover, we note that the oscillation period of two-boson circuit simulator ($T_B \approx \frac{1}{\Delta f_B} = 0.885ms$) is nearly half of that for two pseudofermions ($T_f \approx \frac{1}{\Delta f_f} = 1.77ms$). Compared to the results with $C_F = 0.5C$ (in Fig. 2), we find that the smaller the external force is, the larger the oscillation period and amplitude become, which is consistent with the two-anyon lattice model discussed above.

Finally, we perform circuit simulations of BOs under high-frequency excitations. The time-dependent evolution of pseudospin $|V_{\downarrow,[m,n]}(t)|^2$ at each node in the 2D circuit simulator with $C_F = 0.3C$ and $C_F = 0.5C$ is shown in Supplementary Figures 8a and 8b, where the associated excitation frequency is 2MHz. Other parameters are set as $C_e = 2nF$, $C = 10pF$, and $L = 10uH$. Compared to the associated results with lower excitation frequencies (in Fig. 2 for $C_F = 0.5C$ and in Supplementary Fig. 6 for $C_F = 0.3C$), we find that a more symmetric BO could be realized under a high-frequency excitation.

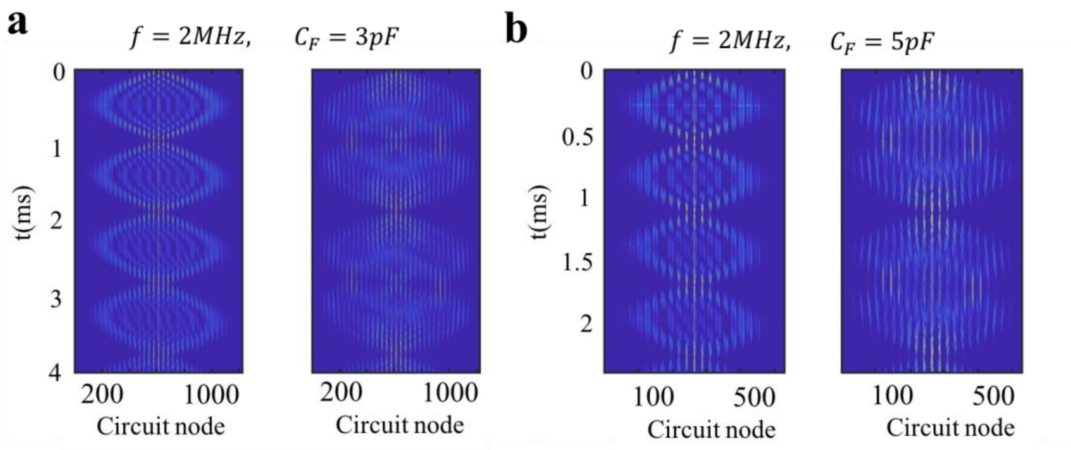

Supplementary Figure 8. Simulated results of Bloch oscillation with different excitation frequencies and values of C_F. The time-dependent evolution of pseudospin $|V_{\downarrow,[m,n]}(t)|^2$ at each node in the circuit simulator with $C_F = 0.3C$ for (a) and $C_F = 0.5C$ for (b). The associated excitation

frequency is 2 MHz.

Supplementary Note 6. The influence of disorder effects on the Bloch oscillations and required phase distributions in circuit simulators. In this part, we have numerically investigated the influence of disorder effects on the BOs and voltage phase distributions in two-boson and two-pseudofermion circuit simulators. As shown in Supplementary Figure 9, the voltage dynamics in two designed electric circuits with different fluctuations of circuit elements (0.5%, 1%, 3% and 5%) are presented. We can see that the period of BOs is still maintained with the small disorder strength. While, when the disorder strength approaches to 3%, the BOs are destroyed for both two-boson and two-pseudofermion circuit simulators. Moreover, the phase distributions of voltage signal at a fixed time (the half in the first BO period) but different disorder strengths are also displayed in Supplementary Figure 10 with the fluctuations of circuit elements being set as 0.5%, 1%, 3% and 5%. In this case, it is shown that the symmetric and antisymmetric distributions of the voltage signal in two-boson and two-pseudofermion circuit simulators are significantly broken when the disorder strength reaches to 3%, being consistent with the BO behavior in Supplementary Figure 9.

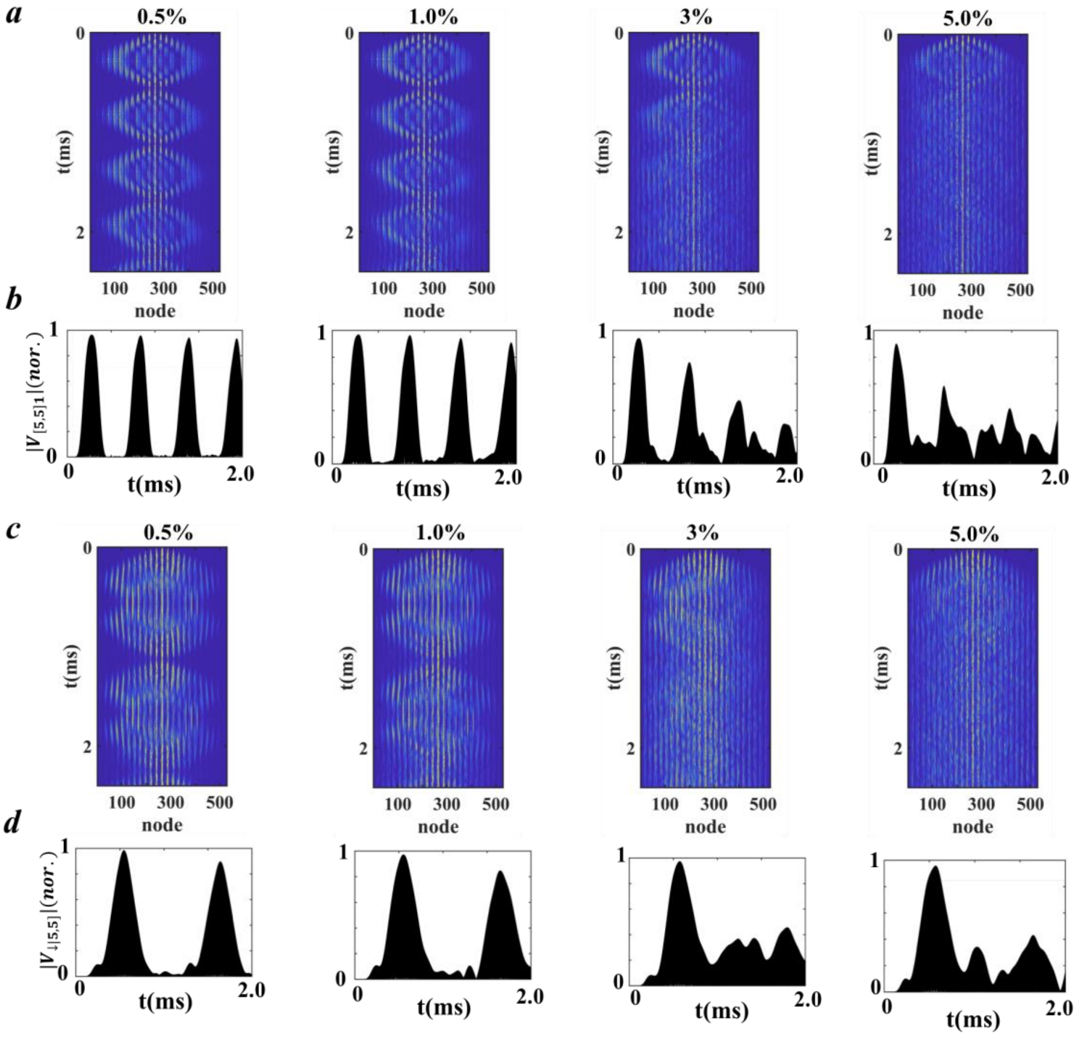

Supplementary Figure 9. Numerical results for the influence of disorder effects on the voltage dynamics. (a) and (c) The voltage dynamics of all nodes in the two-boson and two-pseudofermion circuit simulators with fluctuations of circuit elements being 0.5%, 1%, 3% and 5%. (b) and (d) The voltage amplitude at the (5,5) node in the two-boson and two-pseudofermion circuit simulators with fluctuations of circuit elements being 0.5%, 1%, 3% and 5%.

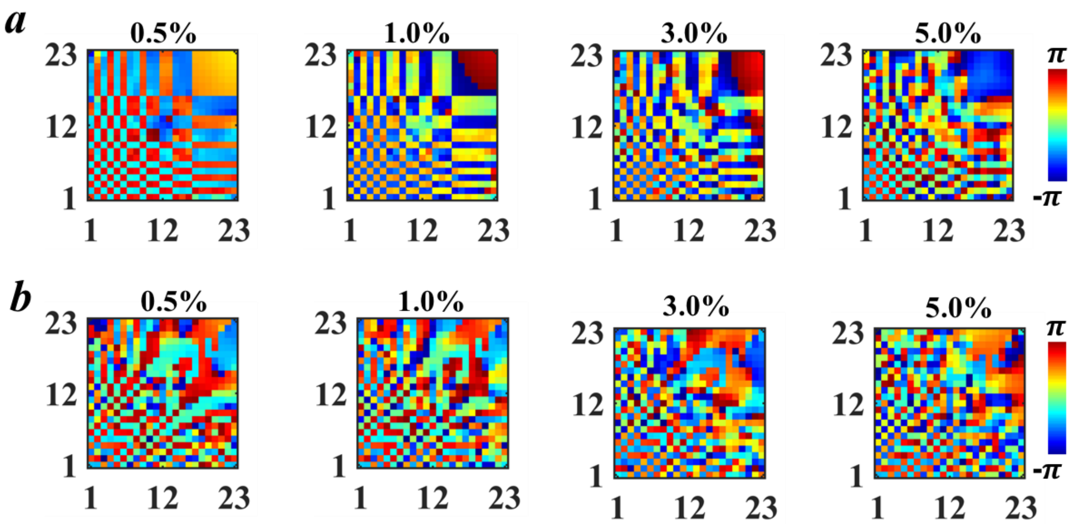

Supplementary Figure 10. Numerical results for the influence of disorder effects on the voltage phase distributions. (a) and (b) The phase distributions of voltage signal at a fixed time (the half in the first BO period) under different disorder strengths 0.5%, 1%, 3% and 5% for two-boson and two-pseudofermion circuit simulators.

Supplementary Note 7. The Influence of loss effect on the simulation of anyonic Bloch oscillations by designed circuit simulators. To quantitatively estimate the loss of our circuit samples, we calculate the voltage dynamics of all nodes in the designed circuit with the effective series resistances of inductance being 10 $m\Omega$, 20 $m\Omega$, 50 $m\Omega$, 70 $m\Omega$ and 100 $m\Omega$, as shown in Supplementary Figure 11a for the two-boson simulator and Supplementary Figure 11c for the two-pseudofermion simulator. The corresponding single-node amplitudes at (5,5) are plotted in Supplementary Figures 11b and 11d, respectively. It is shown that with the series resistances of inductance being increased, the oscillated amplitude is significantly damped. Compared to the experimental results (in Figs. 3f and 3i), we can deduce that the effective series resistance of inductance in the fabricated circuit sample is approximately 50$m\Omega$.

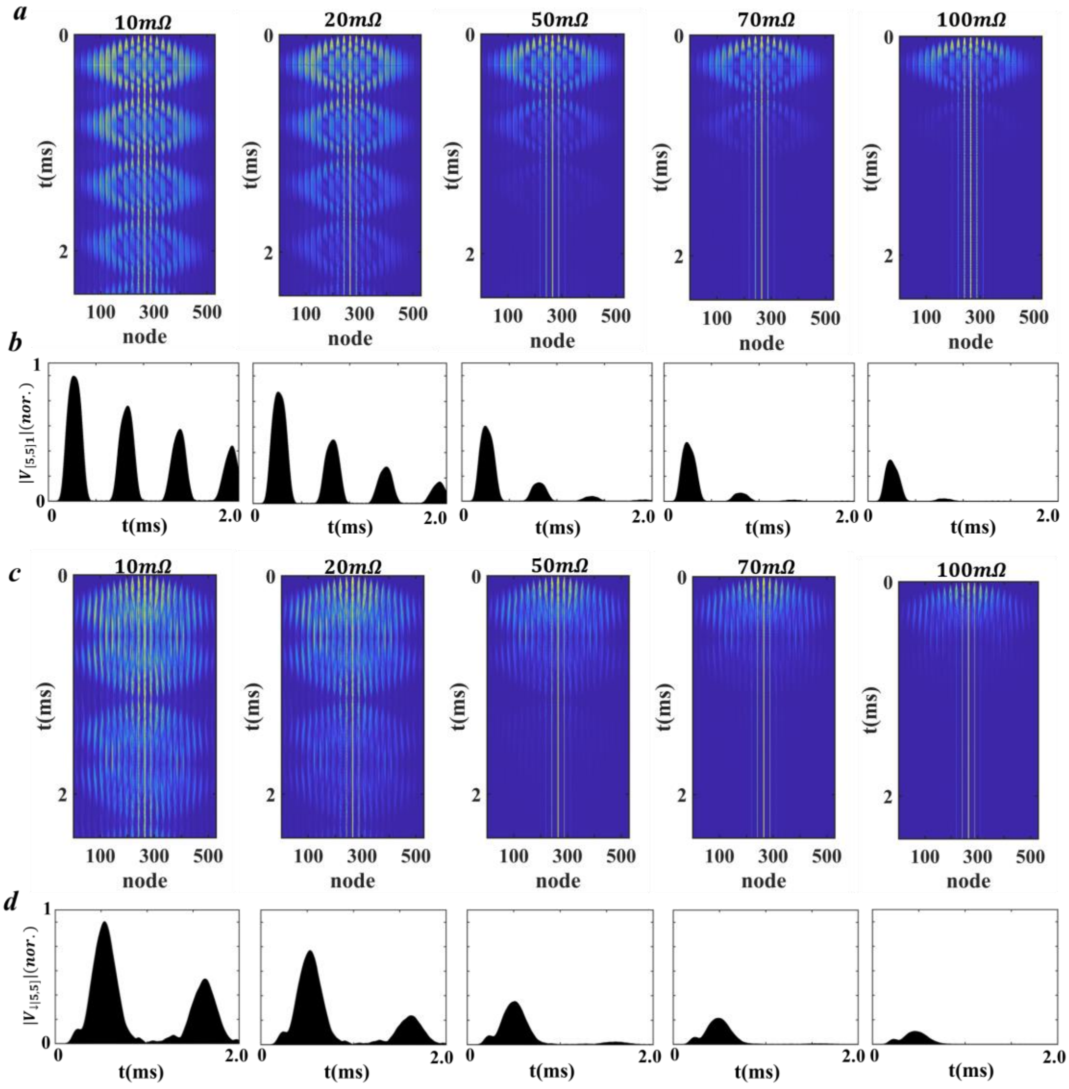

Supplementary Figure 11. Numerical results for the influence of lossy effects on the voltage dynamics. (a) and (b) The voltage dynamics of all nodes in the two-boson and two-pseudofermion circuit simulators with different losses. (c) and (d) The voltage amplitude at the (5,5) node in the two-boson and two-pseudofermion circuit simulators with different losses.

Supplementary Note 8. Numerical results of Bloch oscillations based on the 1D anyonic lattice model with dissipations. To clarify the influence of losses on the BO, we extend the original two-anyon lattice model to contain the intrinsic dissipation rate. In this case, the original two-anyon model can be rewritten as:

$$H = -J\sum_{l=1}^{N}(a_l^{+}a_{l+1} + a_{l+1}^{+}a_l) + i\sum_{l=1}^{N}\gamma n_l + F\sum_{l=1}^{N} l n_l. \quad (15)$$

Based on such a non-Hermitian model, we give numerical results of BOs for two bosons and two pseudofermions. Here, we set γ=0.1, and other parameters are the same as those used in Fig. S2 of the Supplementary Materials. As shown in Supplementary Figures 12a and 12b, we calculate the evolution

of $|c_{mn}(t)|$ with $\theta = 0$ and $\theta = \pi$. Moreover, Supplementary Figures 12c and 12d display the evolution of a fixed state $|c_{5,5}(t)|$ with $\theta = 0$ and $\theta = \pi$, respectively. It is clearly shown that the damped periodic dynamics of both bosons and pseudofermions appear, and the oscillation period of the two bosons is still twice of that for the two pseudofermions. Such damped BOs are consistent to the measured result in circuits. In this case, losses in the fabricated circuit could be mapped to the dissipation rate of the 1D anyonic lattice model.

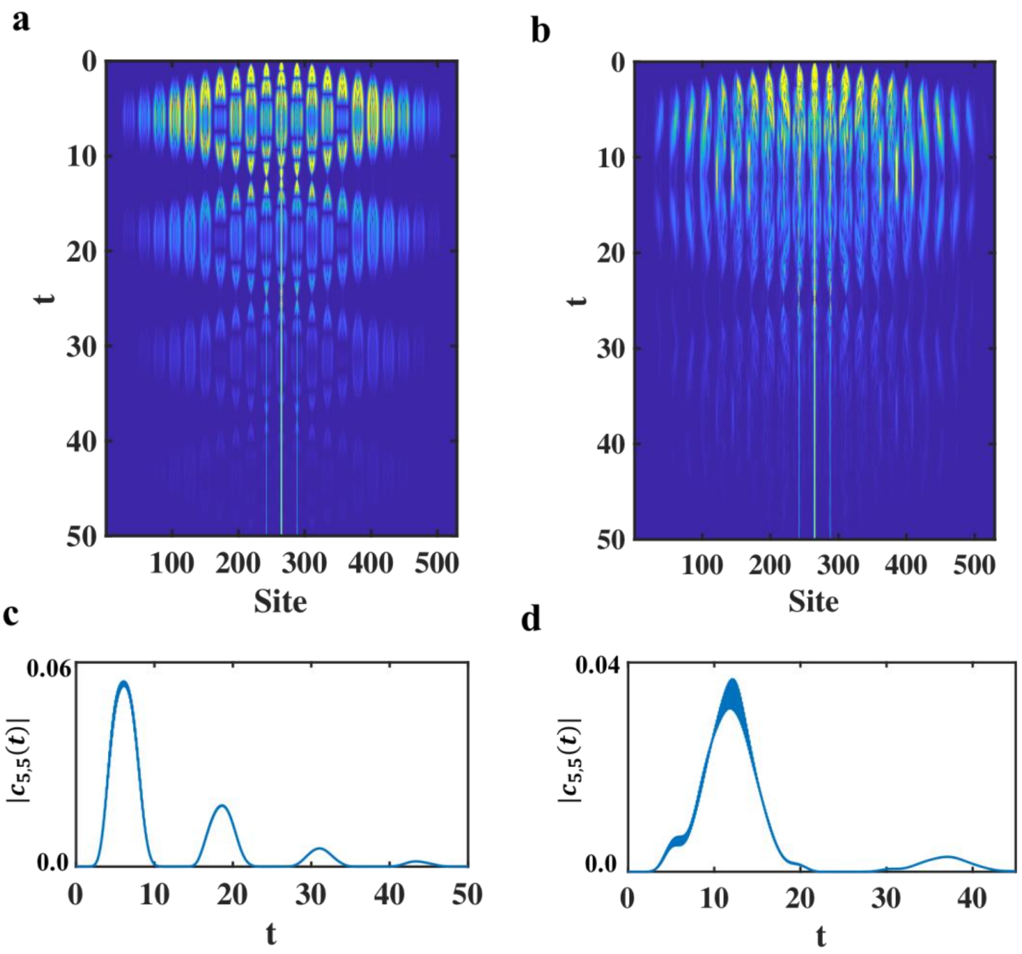

Supplementary Figure 12. Numerical results of Bloch oscillations based on the 1D anyonic lattice model with dissipations. (a) and (b) The evolution of two-anyon state $|c_{mn}(t)|$ under the intrinsic dissipation rate with $\theta = 0$ and $\theta = \pi$. (c) and (d) The evolution of a fixed state $|c_{5,5}(t)|$ with $\theta = 0$ and $\theta = \pi$.

Supplementary Note 9. The precise correspondence between the time-dependent Schrödinger equation of two bosons and two pseudofermions and the designed RC circuit simulators. It is worth noting that the stationary eigenequation of our designed LC circuit is consistent with the stationary

Schrödinger equation of the 1D anyon-Hubbard model with two anyons. For the time-dependent evolution equation, the voltage of the LC circuit follows a second-order time differential, which is different from the first-order time differential of quantum wave functions. In this section, we design another kind of electric circuit based on resistances and capacitances to precisely match the time-dependent Schrödinger equation of two anyons with $\theta = 0$ and $\theta = \pi$.

The designed RC circuit simulator with $\theta = 0$ is plotted in Supplementary Fig. 13a. Here, the associated 1D lattice length is N. We note that the designed circuit simulator contains $2N^2$ nodes, where the row (column) of N^2 nodes is labeled by r=(1, 1),…,(N, N) [c=(N+1,N+1),…,(2N, 2N)]. The voltages of the N^2 circuit node in the top row (left column) correspond to the (copy of) probability amplitudes of two anyons with $\theta = 0$ in the 1D lattice of N sites. Specifically, the probability amplitude of two-boson states c_{mn} is mapped to the voltage signal on the circuit node (m, n) as $V_{m,n}$. Each node is connected to an external DC through a switch to apply an initial voltage signal. Two nodes (one from the row and the other from the column) are connected by suitably designed negative impedance converters with current inversion (INICs), named R_{rc}, to realize hopping, on-site interactions and external forcing. Specifically, the designed INIDs for realizing the particle hoppling rate ($R_{rc} = R_J$) and the external force ($R_{rc} = R_F$/(m+n)) are enclosed by yellow and red blocks, respectively. Here, we set the on-site interaction as zero. For the grounding, the green (blue) circuit node in the row (column) is grounded with a constant capacitor C and an INIC (normal resistor) with the effective resistance being R_{r0} (R_{c0}). In this case, the effective hoppling rate between nodes r=(m, n) and c=(m', n') is $J = \frac{1}{CR_J}$, where the node locations should satisfy the relation of $n' = n \pm 1 + N$ and $m' = m + N$ or $n' = n + N$ and $m' = m \pm 1 + N$. The external force could be mapped to the position-dependent grounding $R_F/(m + n)$ with r=(m,n) and c=(m+N,n+N). In this case, the effective external force is $F = \frac{1}{CR_F}$. The detailed node connections are plotted in the bottom right part of Supplementary Figure 13a.

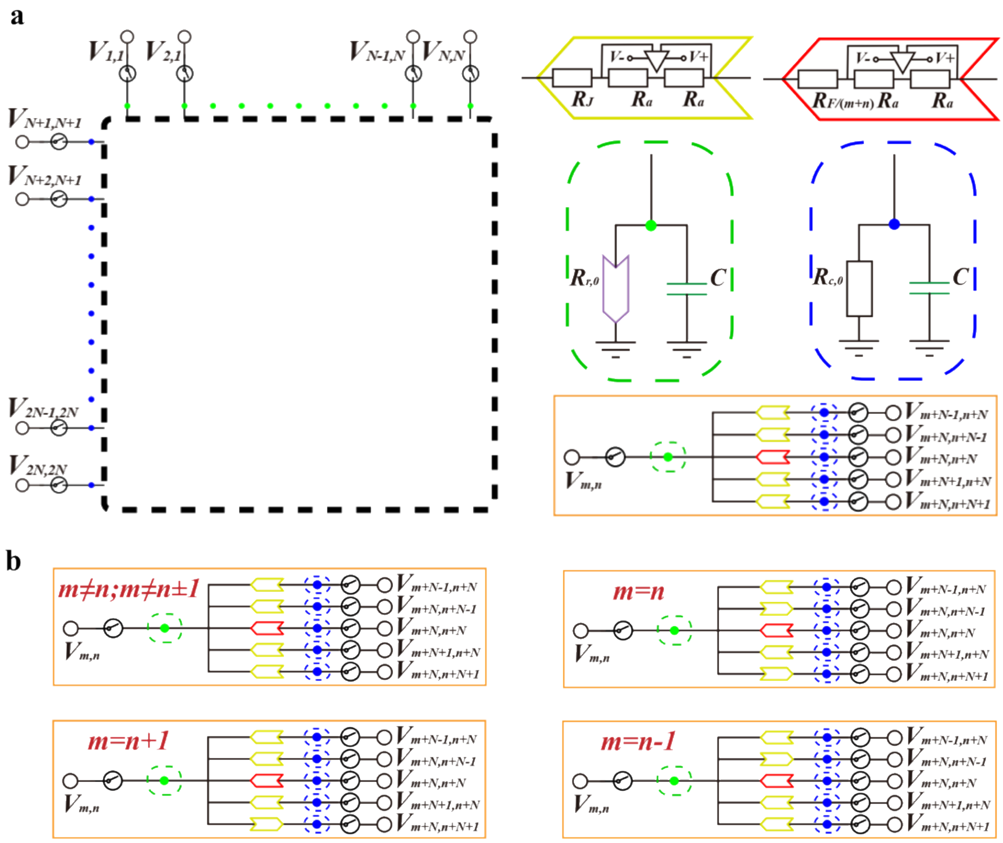

Supplementary Figure 13. The designed RC circuit for simulating anyonic Bloch oscillations. (a) and (b) The designed *RC* circuit for simulating Bloch oscillations of two bosons and two pseudofermions.

By switching off all switches at the same time (applying an initial state), the evolution of voltage at each circuit node can be derived based on the time-dependent Kirchhoff equation as:

$$C\frac{dV_r}{dt} - \frac{V_r}{R_{r0}} = \sum_c \frac{V_c - V_r}{R_{rc}},$$

$$C\frac{dV_c}{dt} + \frac{V_c}{R_{c0}} = \sum_r \frac{V_r - V_c}{-R_{rc}} \tag{16}$$

where V_r (V_c) is the voltage at the circuit node in the row (column). The summation is limited to the connected circuit nodes. Defining the voltages at all circuit nodes as $|V(t)>=[V_{(1,1)}(t),\dots,V_{(N,N)}(t),V_{(N+1,N+1)}(t),\dots,V_{(2N,2N)}(t)]$, Supplementary Eq. (16) can be expressed in matrix form as $i\partial_t|V(t)>=\Xi|V(t)>$, where the off-diagonal components of circuit Hamiltonian Ξ are $\Xi_{rc}=i\frac{1}{CR_{rc}}$ and $\Xi_{cr}=-i\frac{1}{CR_{rc}}$, and the diagonal components are given by $\Xi_{rr}=i\frac{1}{C}(\frac{1}{R_{r0}}-\sum_c\frac{1}{R_{rc}})$ and $\Xi_{cc}=i\frac{1}{C}(-\frac{1}{R_{c0}}+\sum_r\frac{1}{R_{rc}})$. By appropriately setting the grounding INICs as $\frac{1}{R_{r0(i)}}=\sum_k\frac{1}{R_{ik}}$ and the grounding resistances as $\frac{1}{R_{c0(i)}}=\sum_k\frac{1}{R_{ik}}$, the circuit Hamiltonian can be expressed as:

$$\Xi = i\begin{vmatrix} 0 & -\Pi \\ \Pi & 0 \end{vmatrix} \quad (17)$$

with Π being a $N \times N$ matrix. In this case, when the nodes connecting and grounding resistances are suitably applied, the form of the $N \times N$ matrix Π can be the same as the Hamiltonian of the 1D two-boson model. In this case, the voltage evolution in the designed RC circuit could be the same as the probability amplitude of the two bosons.

Based on a similar method, an RC circuit related to two pseudofermions $\theta = \pi$ could also be designed. Supplementary Figure 13b presents the corresponding connection pattern at different circuit nodes. Compared to the circuit for two bosons, the only difference is that there are a few effective hopping rates sustaining a phase $e^{i\pi}$. This could be easily fulfilled by reversing the biased voltage of the associated grounding and connecting INICs.

Then, we use the designed *RC* circuit to simulate the BOs of two bosons and two pseudofermions. Other parameters are set as *N*=35, C=1 μF, $R_J = 1000\Omega$, $R_F = 2000\Omega$ and $R_a = 100\Omega$. The initial voltage distribution is set as $V_{mn}(t=0) = V_0\delta_{12,12}$. As shown in Supplementary Figures 14a and 14b, we present the calculated evolution of the signal $|V_{\downarrow,[m,n]}(t)|^2$ in the circuit simulators for two bosons and two pseudofermions. The corresponding time-dependent evolutions of $|V_{\downarrow,[12,12]}|^2$ are presented in Supplementary Figures 14c and 14d.

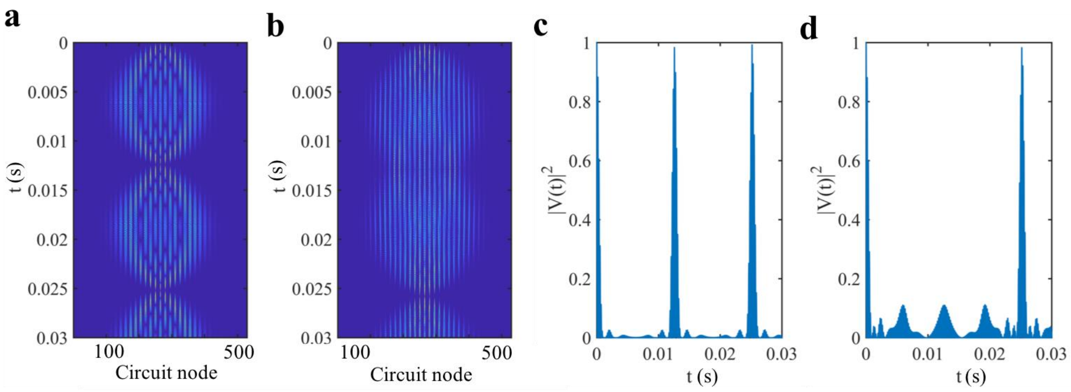

Supplementary Figure S14. Numerical results of the designed RC circuit for simulating anyonic Bloch oscillations. (a) and (b) The evolution of the signal $|V_{\downarrow,[m,n]}(t)|^2$ in the circuit simulators for two bosons and two pseudofermions, respectively. (c) and (d) The time-dependent evolutions of $|V_{\downarrow,[12,12]}|^2$ for two bosons and two pseudofermions.

For comparison, we also calculate the evolution of $|c_{mn}(t)|^2$ of two bosons and two

pseudofermions in the 1D anyon-Hubbard model, as shown in Supplementary Figures 15a and 15b. The associated parameters are set as J=1, F=0.5 and $C_{mn}(t=0)=\delta_{12,12}$. The corresponding time-dependent evolutions of $|c_{12,12}(t)|$ are presented in Supplementary Figures 15c and 15d. We note that good agreement for the time-dependent evolution of voltages and probability amplitude is obtained. In particular, it is clearly shown that the oscillation period in the two-boson simulator is twice that in the two-pseudofermion simulator, which is consistent with the theoretical prediction.

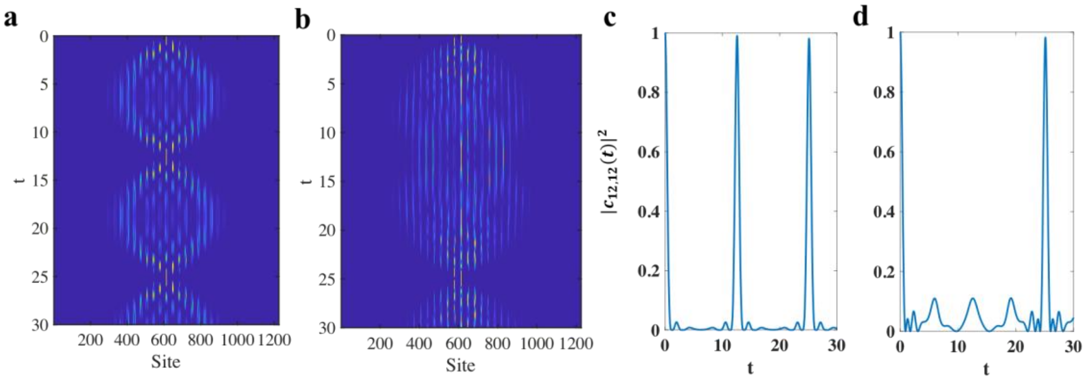

Supplementary Figure 15. Numerical results of the Bloch oscillations for two anyons with an initial state. (a) and (b) The evolution of $|c_{mn}(t)|^2$ for two bosons and two pseudofermions in the 1D anyon-Hubbard model. (c) and (d) The time-dependent evolutions of $|c_{12,12}(t)|$ for two bosons and two pseudofermions.

Supplementary Note 10. Numerical results of anyonic Bloch oscillations beyond bosons and pseudofermions. In this part, we want to demonstrate that the near-perfect Wannier Stark spectrum could also appear at other statistical angle under a suitable value of *F/J* beyond bosons and pseudofermions. For example, as shown in Supplementary Figure 16a, a near-perfect Wannier Stark spectrum could appear at $\theta=1.16$ with *F/J*=1.195, where the energy spacing is 1/3 of that for two bosons, making the BO period of two anyons with $\theta=1.16$ become three times of that for two noninteracting bosons. To illustrate such a novel anyonic BOs, we calculate the evolution of the probability amplitude of two anyons with $\theta=1.16$, as shown in Supplementary Figures 16b and 16c. Here, the lattice size is set as N=15, and the input two-anyon state is $c_{8,8}(t)=e^{i20t}$. For comparison, the evolution results of two bosons are presented in Supplementary Figures 16d and 16e. It is clearly shown that the period of the anyonic BO with $\theta=1.16$ is three times of that for two bosons, being consistent with the calculated Wannier Stark spectra. In addition, we note that the appearance of multiplication period of BOs is not a coincidence. As for another example, if the external forcing is changed to *F/J*=1, the near-perfect

Wannier-Stark spectrum could also appear with the statistical angle being around $\theta = 2.09$, as shown in Supplementary Figure 16f. From the above results, we deduce that the appearance of Wannier Stark spectrum with *F/J*>0.5 should result from a balance between the quantum statistic (θ) and the ratio of external force to the hopping strength (*F/J*). These two factors could collectively control the splitting and coupling of anyonic energy bands. In this case, the Wannier Stark spectrum may appear at suitable values of statistic angle and an external forcing. The much lower spatial symmetry of mapped lattice model of two anyons (beyond bosons and pseudofermions) leads to a smaller energy degeneracy and a denser distribution of eigen-spectrum. Because, the statistical angle does not satisfy the relationship of $\theta = \frac{v}{o}\pi$ (v and o are integers). Hence, realizing the statistical angle $\theta = 1.16$ induced complex couplings by braiding the connection pattern of o adjacent circuit nodes in a single lattice site is unfeasible. Such a problem could be solved by suitably designed *RLC* circuits combined with a negative impedance converter with current inversion [72].

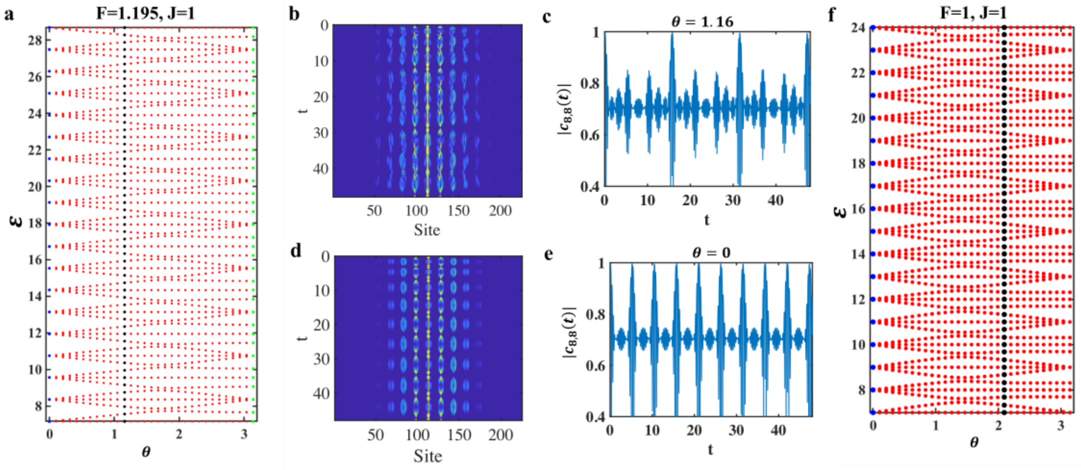

Supplementary Figure 16. Anyonic Bloch oscillations beyond bosons and pseudofermions. (a) Calculated eigen-energies of two anyons as a function of the statistical angle θ with F/J=1.195. (b) and (d) The calculated evolution of the probability amplitude of two anyons with $\theta = 1.16$ and $\theta = 0$. (c) and (e) The evolution results of $|c_{8,8}(t)|$ with $\theta = 1.16$ and $\theta = 0$. Here, the lattice size and external force are set as N=15 and *F/J*=1.195, and the input two-anyon state is $c_{8,8}(t) = e^{i20t}$. (f) Calculated eigen-energies of two anyons as a function of the statistical angle θ with *F/J*=1.
